# Bifidobacterium animalis subsp. lactis BB-12 attenuates diabetic retinopathy through gut microbiota modulation: evidence for the gut–retinal axis

**DOI:** 10.3389/fcimb.2025.1681943

**Published:** 2025-11-06

**Authors:** Jiayi Lin, Yaqi Cheng, Yumin Ling, Simin Gu, Haocheng Zhu, Meng Li, Huan Yu, Jianbing Li, Qian Luo, Weihua Li, Shiqi Ling

**Affiliations:** 1 Department of Ophthalmology, The Third Affiliated Hospital of Sun Yat-sen University, Sun Yat-sen University, Guangzhou, China; 2 State Key Laboratory of Ophthalmology, Zhongshan Ophthalmic Center, Guangdong Provincial Key Laboratory of Ophthalmology and Visual Science, Sun Yat-sen University, Guangzhou, China

**Keywords:** intestinal microbiota, diabetes, gut-retina axis, probiotics, retinopathy

## Abstract

**Purpose:**

Previous studies have shown that *Bifidobacterium animalis subsp. lactis BB-12* plays a role in maintaining the intestinal barrier and regulating inflammation; however, its potential connection to ocular diseases has not been thoroughly explored. Diabetic retinopathy (DR) is a common ocular complication of diabetes and is closely associated with metabolic dysregulation. This study investigated whether BB-12 supplementation could affect systemic diabetic symptoms, the progression of DR, and the stability of gut microbiota.

**Materials and methods:**

Diabetic db/db mice were utilized to monitor metabolic parameters, assess hepatic and lipid profiles, evaluate retinal function via ERG, and examine retinal morphology through OCT and HE staining. Treg/Th17 balance was analyzed by flow cytometry, and gut microbiota composition was profiled using 16S rRNA sequencing.

**Results:**

The results showed that BB-12 reduced obesity, decreased hepatic steatosis, improved retinal blood vessel health and vision, and influenced both the Treg/Th17 balance and gut dysbiosis in diabetic mice.

**Conclusions:**

These findings lay the groundwork for regulatory role of intestinal microbiota on systemic and ocular complications of diabetes, and further examination of the gut-retina axis.

## 1 Introduction

As global living standards rise, diabetes mellitus (DM) has become a significant public health concern due to its systemic complications (American Diabetes Association, 2013). Ocular complications, particularly diabetic retinopathy (DR), pose a significant clinical challenge. While early-stage DR may respond to retinal photocoagulation or intravitreal therapy, advanced tractional retinal detachment often carries a poor prognosis and, in severe cases, may lead to phthisis bulbi due to missed surgical windows (Brown et al., 2021). The eye is a relatively isolated and enclosed organ, protected by the blood–aqueous barrier (BAB) in the anterior segment and the blood–retinal barrier (BRB) in the posterior segment. These anatomical and physiological barriers make drug delivery for DR particularly challenging (Qin et al., 2025). The current standard therapy involves intravitreal injection of anti-VEGF or anti-Ang 2 agents, which must traverse the inner limiting membrane to reach the retina. However, repeated invasive procedures increase the risk of endophthalmitis and vitreous hemorrhage. Owing to the restrictive nature of the BRB, systemically administered drugs have limited retinal bioavailability, and no effective systemic therapies targeting DR are currently available (Hashida and Nishida, 2023).

Recent studies underscore the pivotal role of the gut microbiota in maintaining systemic homeostasis. It activates type I interferon-mediated antiviral immunity via the cGAS-STING pathway (Erttmann et al., 2022) and cooperates with mucosa-associated lymphoid tissue (MALT) to promote adaptive immune responses through pathogen recognition, innate immune activation, and antigen presentation (Jiao et al., 2020). Through modulation of immune responses, regulation of inflammatory mediators, and correction of metabolic imbalances, the gut microbiome can influence disease progression. Altering microbial composition has been linked to improvements in chronic conditions, including obesity and neuropsychiatric disorders (Barrio et al., 2022; Min et al., 2024). The extensive functional capacity and largely unelucidated mechanisms of the gut microbiota remain active areas of investigation.

Following the concepts of the gut-brain axis and the gut-liver axis, the notion of a gut-retina axis has gradually gained recognition (Parker et al., 2022). Our previous studies suggested a potential genetic susceptibility link between intestinal disorders and DR. Gut microbiota dysbiosis has been implicated in the development of ocular diseases. In murine models of age-related macular degeneration (AMD), increased abundances of Clostridium and Bacillus have been observed (Rowan and Taylor, 2018), whereas human AMD patients show elevated levels of Proteobacteria and Bacteroidota (Zhang Y. et al., 2023). In the DR population, genera such as Blautia, Bacteroides, and Megamonas have shown potential as microbial biomarkers (Huang et al., 2021; Bai et al., 2022). The mechanisms underlying the gut-retina axis involve systemic and local inflammatory responses, regulation of T-cell differentiation by short-chain fatty acids (e.g., butyrate), impaired intestinal barrier function leading to endotoxemia, metabolic-induced oxidative stress, and genetic and epigenetic modulation (Kammoun et al., 2024). However, direct experimental evidence connecting gut microbiota modulation to DR remains limited. Among Bifidobacteria, *Bifidobacterium animalis subsp. lactis Bb-12* has demonstrated immunomodulatory effects that mitigate disease progression in various inflammatory conditions (Merenstein et al., 2015; Nocerino et al., 2020). Its regulatory mechanisms may involve lipid metabolism, immune cell populations, and the release of anti-inflammatory mediators (Tonucci et al., 2017). Emerging evidence suggests that DR should be considered an inflammatory rather than a purely vascular disease (Semeraro et al., 2019). Nevertheless, whether modulation of the gut microbiota can effectively ameliorate DR has yet to be definitively established.

**Figure 1:**
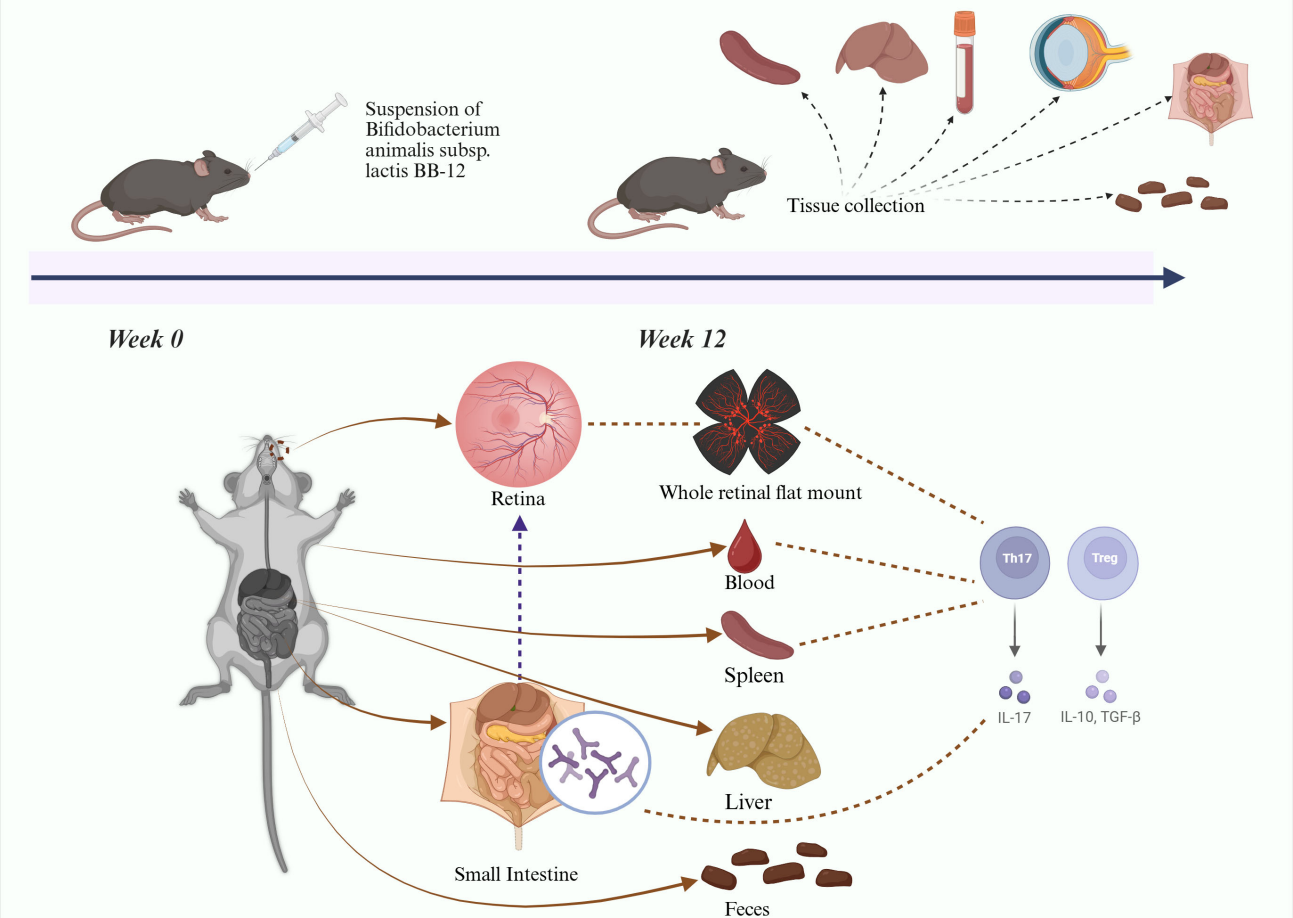
Experimental flow chart.

We hypothesize that oral administration of *Bifidobacterium animalis subsp. lactis BB-12* can modulate gut microbiota composition, regulate systemic and local inflammation, and thereby attenuate the progression of DR in type 2 diabetic mice. Current diabetic mouse models include chemically induced, diet-induced, and genetically modified models. Chemically induced models cause rapid pancreatic cells destruction through cytotoxic agents; however, blood glucose levels may return to normal after drug metabolism. Diet-induced models exhibit mild diabetic phenotypes, making it difficult to reproduce advanced stages of DR. The db/db mouse, a genetically modified model with a leptin receptor mutation, develops persistent hyperglycemia and chronic insulin resistance, closely resembling the progression of human type 2 diabetes. Therefore, db/db mice were selected for this study (Azushima et al., 2018). Diabetic mice were orally administered *Bifidobacterium animalis subsp. lactis Bb-12* to evaluate systemic and tissue-specific effects (**Figure 1**), aiming to elucidate the impact of gut microbiota modulation on immune-privileged organs and further support the gut–retina axis concept, to identify potential therapeutic strategies for DR. (**Figure 1** about here).

## 2 Materials and methods

### 2.1 Animal experiment and study design

Male C57BLKS/J db/db mice with spontaneous mutations in the leptin receptor and their non-diabetic db/m littermates (SPF grade; 6 weeks old; Jiangsu Jicui Laboratory Animal Co., Ltd.) were randomly divided into three groups (n=9/group): Control group, T2D group, and Bb-12 group. The Control group consisted of male C57BLKS/J db/m heterozygous littermates of db/db mice, serving as non-diabetic controls. The T2D group included homozygous db/db mice with leptin receptor deficiency. The Bb-12 group received a daily oral gavage of 0.1 mL of *Bifidobacterium animalis subsp. lactis Bb-12* probiotic suspension (purchased from Shanghai Difule Biotechnology Co., Ltd.; 1.9 × 10^10^ CFU/mL).

All experimental procedures were conducted in strict accordance with the ARVO Animal Statement and were approved by the Institutional Animal Care and Use Committee of the Third Affiliated Hospital of Sun Yat-sen University. Mice were housed in individually ventilated cages under standard conditions (temperature 22 ± 1°C, humidity 50 ± 5%) with ad libitum access to food and water. The experimental period lasted 12 weeks, during which fasting glucose and body weight were recorded on a biweekly basis.

When animal anesthesia was required during the experiment, all procedures adhered to the *AVMA Guidelines for the Euthanasia of Animals: 2020 Edition* and conformed to the principles of the 3Rs. Animals were anesthetized via intraperitoneal injection of sodium pentobarbital (40 mg/kg) prior to examinations. During experimental procedures, body temperature was maintained with a heating pad, and corneal surfaces were kept moist to prevent drying. For tissue harvesting, euthanasia was induced by an intraperitoneal overdose of sodium pentobarbital (120 mg/kg), followed by cervical dislocation to ensure death, after which carcasses were disposed of in a biohazard-safe manner.

### 2.2 Retina optic coherence tomography

At week 12, the mice were anesthetized with an intraperitoneal injection of sodium pentobarbital (40 mg/kg). Pupillary dilation was induced using compound tropicamide eye drops. Once adequate mydriasis was confirmed, retinal OCT imaging was performed with equipment from Heidelberg Engineering (Germany). Fundus photographs and OCT images were obtained, and retinal layer analysis was performed using ImageJ software.

### 2.3 Electroretinography

At week 12, retinal ERG was performed to evaluate retinal electrophysiological function. The mice underwent a 12-hour period of dark adaptation prior to the procedure. Mice were anesthetized and pupils dilated; corneal electrodes, subcutaneous reference, and ground electrodes were positioned (Chan et al., 2020). Under scotopic conditions, stimuli of 0.0003 cd·s/m², 0.01 cd·s/m², and 3.0 cd·s/m² were sequentially applied. After 5 minutes of light adaptation, a photopic stimulus of 3.0 cd·s/m² was delivered. ERG waveforms were recorded and subsequently analyzed using Spyder (Python 3.12).

### 2.4 Retinal whole mounts

An appropriate amount of Evans Blue powder (Macklin Biochemical, Shanghai) was thoroughly dissolved in sterile saline and then filtered. The Evans Blue solution was administered via tail vein injection. After 1 hour of dye circulation, eyeballs were enucleated and fixed in 4% paraformaldehyde at 4°C for 12 hours, rinsed with PBS, and retinas were dissected under a stereomicroscope. Retinas were carefully flat-mounted onto glass slides and secured with coverslips. Images were acquired using a laser scanning confocal microscope (ZEISS LSM 880). Retinal vascular density was subsequently analyzed using Spyder (Python 3.12).

### 2.5 Hematoxylin-eosin and Periodic acid-Schiff staining

At week 12, the eyeballs, liver, and small intestine were harvested, fixed in 4% paraformaldehyde, embedded in paraffin, sectioned, and subjected to HE staining according to standard histological evaluation protocols. Retinal vasculature was assessed using PAS staining, with morphological analysis performed via CaseViewer software.

### 2.6 Flow cytometry of blood, spleen, and retina

Mice were euthanized via overdose anesthesia, followed by the sample of Blood, spleen, and retina were processed into single-cell suspensions. Cells were stained for surface markers using APC/Cyanine7 CD45 Antibody, PerCP/Cyanine5.5 CD3 Antibody, APC CD25 Antibody, and FITC CD4 Antibody. Following fixation and permeabilization according to the manufacturer’s protocols, intracellular staining was performed using PE FOXP3 Antibody and PE/Cyanine7 IL-17A Antibody (both from BioLegend, USA). Cell analysis was conducted using a Beckman Coulter CytoFLEX flow cytometer, and data were analyzed with FlowJo v11 software.

### 2.7 Retinal quantitative real-time PCR

Mice were euthanized by overdose anesthesia, and eyeballs were enucleated to isolate retinas. Total RNA was extracted following the manufacturer’s protocol using RNA extraction reagent, chloroform substitute, isopropanol, and RNA dissolution buffer. Reverse transcription was performed using a commercial cDNA synthesis kit on a PCR instrument. qPCR was conducted with Universal Blue SYBR Green qPCR Master Mix and specific gene primers (**Table 1**). The amplification protocol consisted of an initial denaturation step at 95°C for 30 seconds, followed by 40 cycles of denaturation at 95°C for 15 seconds and extension at 60°C for 30 seconds, concluding with a melt curve analysis. Relative gene expression was calculated using the ΔΔCT method (Yourick et al., 2019). All reagents were purchased from Wuhan Servicebio Biotechnology.

**Table 1:** Primers used for real-time quantitative PCR.

Gene name	Forward primer	Reverse primer
RORYT	GGATGAGATTGCCCTCTACACG	GCGGCTTGGACCACGATG
IL-17	TCCACCGCAATGAAGACCCT	CATGTGGTGGTCCAGCTTTCC
Foxp3	ATGAGAAAGGCAAGGCCCAGT	GTGGCTACGATGCAGCAAGA
TGF-beta	GCTGAACCAAGGAGACGGAATA	GGCTGATCCCGTTGATTTCC
VEGF	GAGCGTTCACTGTGAGCCTTGT	TTAACTCAAGCTGCCTCGCCT
IL-10	AATAAGCTCCAAGACCAAGGTGT	CATCATGTATGCTTCTATGCAGTTG

### 2.8 Determination of blood biochemical indicators

Serum levels of aminotransferase (AST), alanine aminotransferase (ALT), triglycerides (TG), total cholesterol (TC), low-density lipoprotein cholesterol (LDL-C), and high-density lipoprotein cholesterol (HDL-C) were measured using the CASTS-DL0086 fully automated biochemical analyzer.

### 2.9 Gut microbiome analysis

Microbial DNA from mouse fecal samples was extracted using the E.Z.N.A.^®^ Gel Extraction Kit (Omega Bio-tek, Norcross, GA, USA) following the manufacturer’s protocol. Subsequent library preparation was performed according to the NEBNext^®^ Ultra™ DNA Library Prep Kit for Illumina^®^ standard protocol. The V3–V4 regions of the 16S rRNA gene were PCR-amplified using primers 338F (5′-ACTCCTACGGGAGGCAGCA-3′) and 806R (5′-GGACTACHVGGGTWTCTAAT-3′). Amplicons were purified using NEBNext Sample Purification Beads. Library sequencing was performed by Megagen Biotechnology Co., Ltd. (Shenzhen, China) using the Illumina NovaSeq PE250 platform.

### 2.10 Statistical analysis

Data were analyzed using Excel 2021, GraphPad Prism 10.0 (GraphPad Software, USA), and Spyder (Python 3.12). Normality was assessed using the Shapiro–Wilk test. Parametric data are presented as mean ± SEM and analyzed by one-way ANOVA, while non-parametric data were evaluated using the Kruskal–Wallis H test. Spearman’s rank correlation was used for correlation analysis. A p-value < 0.05 was considered statistically significant.

## 3 Results

### 3.1 Effects of Bifidobacterium animalis subsp. lactis Bb-12 on physiological and serum biochemical parameters in diabetic mice

**Figure 2:**
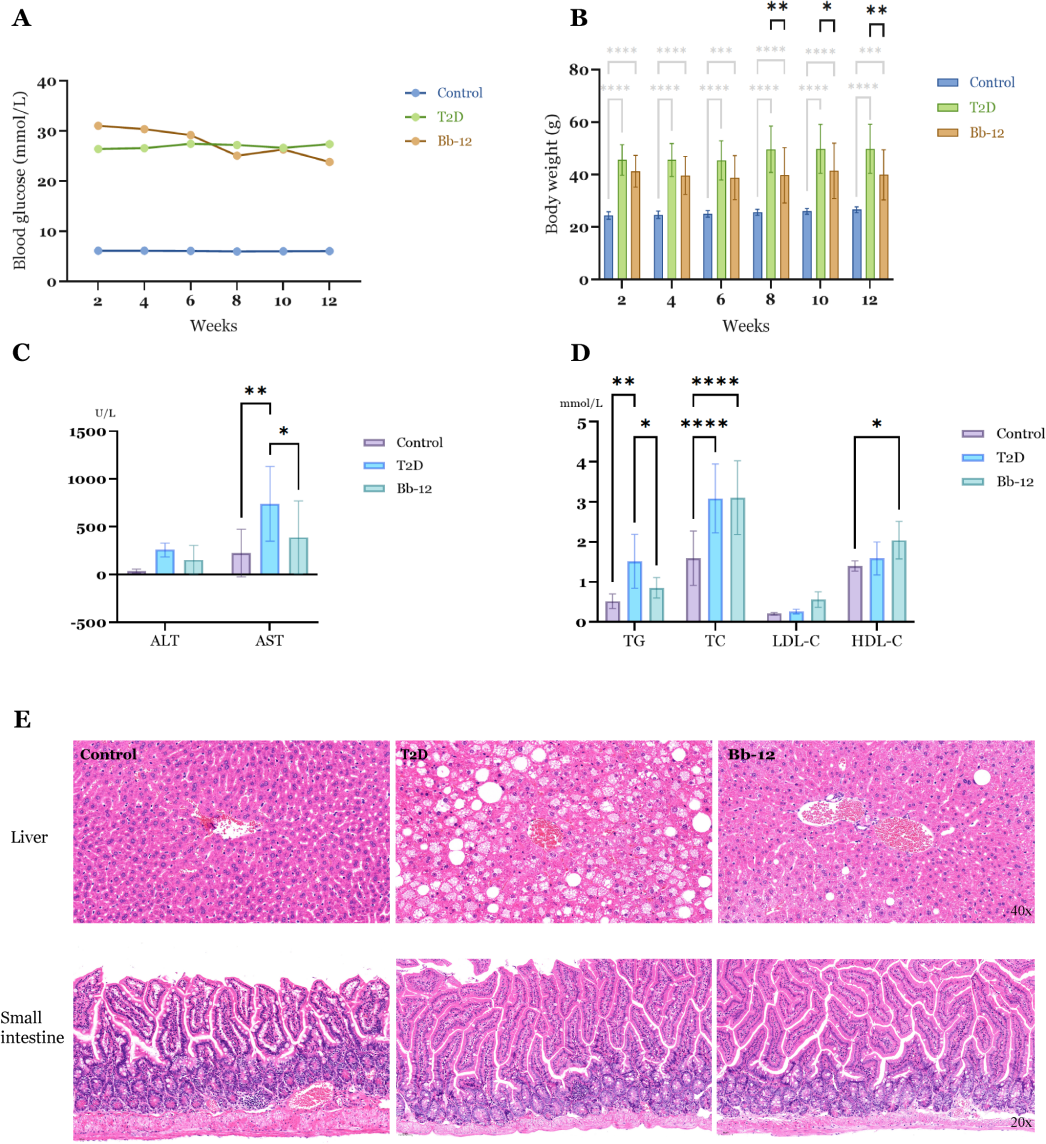
Fasting blood glucose changes **(A)** and body weight changes **(B)** in the three groups during the experiment. From the 8th week, the body weight of the Bb-12 group decreased compared with the T2D group (P < 0.05) (n=9). Liver function indexes **(C)** and blood lipid levels **(D)** in the three groups at the 12th week of the experiment. **(E)** HE staining of liver and small intestine sections from the experimental groups (n=5). *p < 0.05, **p < 0.01, ***P ≤ 0.001 and ****P ≤ 0.0001.

Throughout the experiment, blood glucose levels in the control group remained between 5.0 and 8.0 mmol/L, whereas both the T2D and Bb-12 groups exhibited elevated levels, ranging from 9 to 36 mmol/L. The control group had significantly lower glucose than the T2D and Bb-12 groups (P < 0.0001). Although the mean glucose in the Bb-12 group was lower than that of T2D during weeks 8–12, this difference was not statistically significant (P > 0.5) (**Figure 2A**). Body weight in the control group was consistently lower than in the T2D and Bb-12 groups throughout the study (P < 0.0001). From week 8 onwards, the Bb-12 group showed significantly reduced body weight compared to the T2D group (P < 0.05), accompanied by increased activity levels relative to T2D mice (**Figure 2B**).

Serum analyses revealed elevated ALT levels in the T2D group compared to the control and Bb-12 groups, although the differences were not statistically significant. AST levels were significantly increased in diabetic mice relative to controls, but Bb-12 supplementation attenuated this rise (P < 0.05). TG and TC were markedly higher in diabetic mice than in controls (P < 0.01), with the Bb-12 group showing reduced TG levels compared to T2D mice (P < 0.05). HDL-C was elevated in the Bb-12 group versus controls (P < 0.05), but not significantly different from the T2D group (**Figures 2C, D**).

As shown in **Figure 2**, *Bifidobacterium animalis subsp. lactis Bb-12* supplementation aided in body weight control but did not fully reverse hyperglycemia or obesity in diabetic mice. It also partially alleviated hepatic injury and demonstrated a modulatory effect on dyslipidemia. (**Figure 2** about here)

### 3.2 Bifidobacterium animalis subsp. lactis Bb-12 alleviates hepatic steatosis in diabetic mice

HE staining of liver and small intestine tissues is shown in **Figure 2E**. The control group exhibited well-defined hepatic architecture with tightly arranged hepatocytes and no morphological features of cellular degeneration. In contrast, diabetic mice displayed disorganized hepatic cell arrangement and numerous cytoplasmic lipid droplets of varying sizes. Hepatic steatosis was particularly severe in the T2D group, whereas the Bb-12 group showed notably reduced lipid accumulation compared to T2D mice. Intestinal histology revealed intact intestinal wall and crypt structures across all three groups, with clearly defined Paneth cells and no evident inflammatory cell infiltration or tissue disruption.

### 3.3 Bifidobacterium animalis subsp. lactis Bb-12 delays retinal thickness reduction in diabetic mice

Histologically, the retina is divided into 10 layers from the vitreous to the choroid: internal limiting membrane (ILM), nerve fiber layer (NFL), ganglion cell layer (GCL), inner plexiform layer (IPL), inner nuclear layer (INL), outer plexiform layer (OPL), outer nuclear layer (ONL), external limiting membrane (ELM), inner segment/outer segment layer (IS/OS), and retinal pigment epithelium (RPE). For quantitative analysis, the retina was divided into three regions: inner retina (from ILM to inner INL), middle retina (from outer INL to outer ONL), and outer retina (from ELM to RPE).

**Figure 3:**
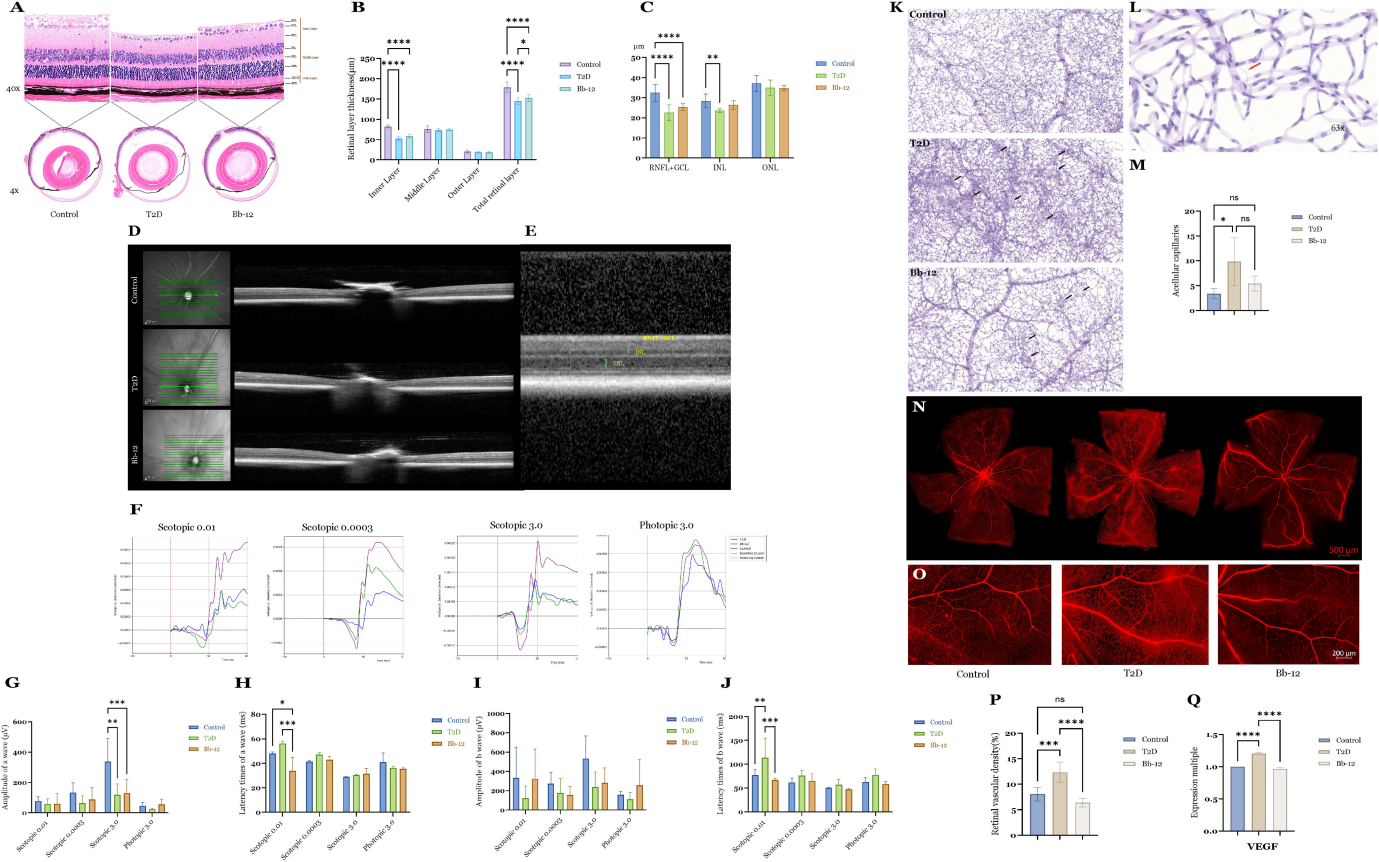
Retinal HE staining images **(A)** (n=4) and layer thickness comparison **(B)** of the three groups; retinal OCT images **(D, E)** and layer thickness comparison **(C)** of the three groups (n=8). ERG recordings from the three experimental groups under scotopic and photopic conditions **(F)** comparative analysis of a-wave amplitudes **(G)** comparison of a-wave latency times **(H)** comparative analysis of b-wave amplitudes **(I)** and comparison of b-wave latency times **(J)**. PAS staining at 20× magnification showing representative microaneurysms in the three groups [**(K)**, black arrows]; typical acellular capillaries at 63× magnification [**(L)**, red arrows] and their quantification among groups **(M)** (n=4); whole-mount retinal flat mounts imaged by confocal microscopy **(N, O)** comparison of retinal vascular density **(P)** (n=4) and VEGF mRNA expression **(Q)** among the groups (n=5).*P < 0.05, **P < 0.01, ***P < 0.001, ****P < 0.0001.

HE staining results (**Figures 3A, B**) demonstrated a significant reduction in total retinal thickness in diabetic mice compared to controls (P < 0.0001). The Bb-12 group exhibited increased total retinal thickness relative to the T2D group (P < 0.05). Inner retinal thickness was markedly decreased in diabetic mice versus controls (P < 0.0001). Although the Bb-12 group showed greater inner retinal thickness than the T2D group, this difference was not statistically significant. No significant differences were observed among the three groups in middle and outer retinal thickness.

OCT images and quantitative analysis (**Figures 3C–E**) focused on layers discernible by this modality, including the combined RNFL+GCL, INL, and ONL layers. Diabetic mice exhibited reduced RNFL+GCL thickness compared to controls (P < 0.0001), with the Bb-12 group showing a non-significant trend towards increased thickness relative to the T2D group. The INL thickness in the T2D group was significantly lower than that of controls (P < 0.01). While the Bb-12 group’s INL thickness was reduced compared to controls, it increased relative to the T2D group, but these differences lacked statistical significance. (**Figure 3** about here)

### 3.4 Effects of Bifidobacterium animalis subsp. lactis Bb-12 on ERG in diabetic mice

ERG was performed to evaluate retinal electrophysiological function. The a-wave primarily reflects photoreceptor activity, representing rod function in scotopic and cone function in photopic conditions. A reduction in a-wave amplitude indicates photoreceptor loss or dysfunction, whereas a prolonged latency reflects delayed photoreceptor responses. Correspondingly, HE staining often reveals a decreased number of cells in the ONL and disorganization of the photoreceptor OS. The b-wave primarily reflects the activity of bipolar and Müller cells; abnormalities in the b-wave are commonly associated with disrupted cellular organization or degenerative changes within the INL on histological examination. On ERG recordings, the a-wave appears as the initial negative deflection, followed by the positive b-wave peak (McAnany et al., 2022). **Figure 3F** displays representative waveforms from the three groups under scotopic stimuli of 0.0003, 0.01, and 3.0 cd·s/m², as well as a photopic stimulus of 3.0 cd·s/m². As shown in **Figures 3G–J**, under scotopic 0.01 cd·s/m² stimulation, the Bb-12 group exhibited significantly reduced peak latencies of both a- and b-waves compared to the T2D group, suggesting that Bb-12 supplementation may enhance retinal signal transmission speed and partially ameliorate diabetes-induced visual dysfunction.

### 3.5 Bifidobacterium animalis subsp. Lactis Bb-12 attenuates retinal vascular pathology in diabetic mice

To evaluate retinal vascular morphology, PAS staining and whole-retina flat-mount preparations were performed. **Figure 3K** shows representative PAS-stained retinal sections from the three groups. Black arrows indicate retinal microaneurysms, characterized by focal capillary outpouchings containing proliferative endothelial cells. Microaneurysm formation in DR is attributed to endothelial dysfunction and basement membrane abnormalities. No microaneurysms were observed in control mice, whereas diabetic mice exhibited abundant microaneurysms. The Bb-12 group demonstrated fewer microaneurysms and reduced vascular density compared to the T2D group. In addition to microaneurysms, acellular capillaries—capillary segments devoid of endothelial and pericyte cells, indicative of microcirculatory impairment—were assessed. **Figure 3L** illustrates a typical acellular capillary. Retinas were divided into 15 uniform units, and the number of acellular capillaries per unit was quantified. The T2D group showed significantly increased acellular capillaries compared to controls (P < 0.05) and a higher count than the Bb-12 group, although the latter difference was not statistically significant (**Figure 3M**).

To evaluate blood–retina barrier integrity, whole-retina flat-mount fluorescence imaging was performed. As shown in **Figures 3O, P**, vascular leakage was significantly higher in the T2D group compared to the control and Bb-12 groups, characterized by punctuate fluorescence accumulation and localized diffusion, primarily in the peripheral retina. Vascular density analysis (**Figure 3Q**) indicated a notable increase in retinal vessel density in T2D mice relative to control and Bb-12 groups (P < 0.001). VEGF mRNA levels in retinal tissues (**Figure 3R**) were also significantly higher in the T2D group compared to the control and Bb-12 groups (P < 0.0001). Overall, these results suggest that *Bifidobacterium animalis subsp. lactis Bb-12* supplementation partially mitigates diabetic retinal vascular damage.

### 3.6 Bifidobacterium animalis subsp. lactis Bb-12 modulates Treg/Th17 balance and reduces retinal Th17 cytokine expression in diabetic mice

**Figure 4:**
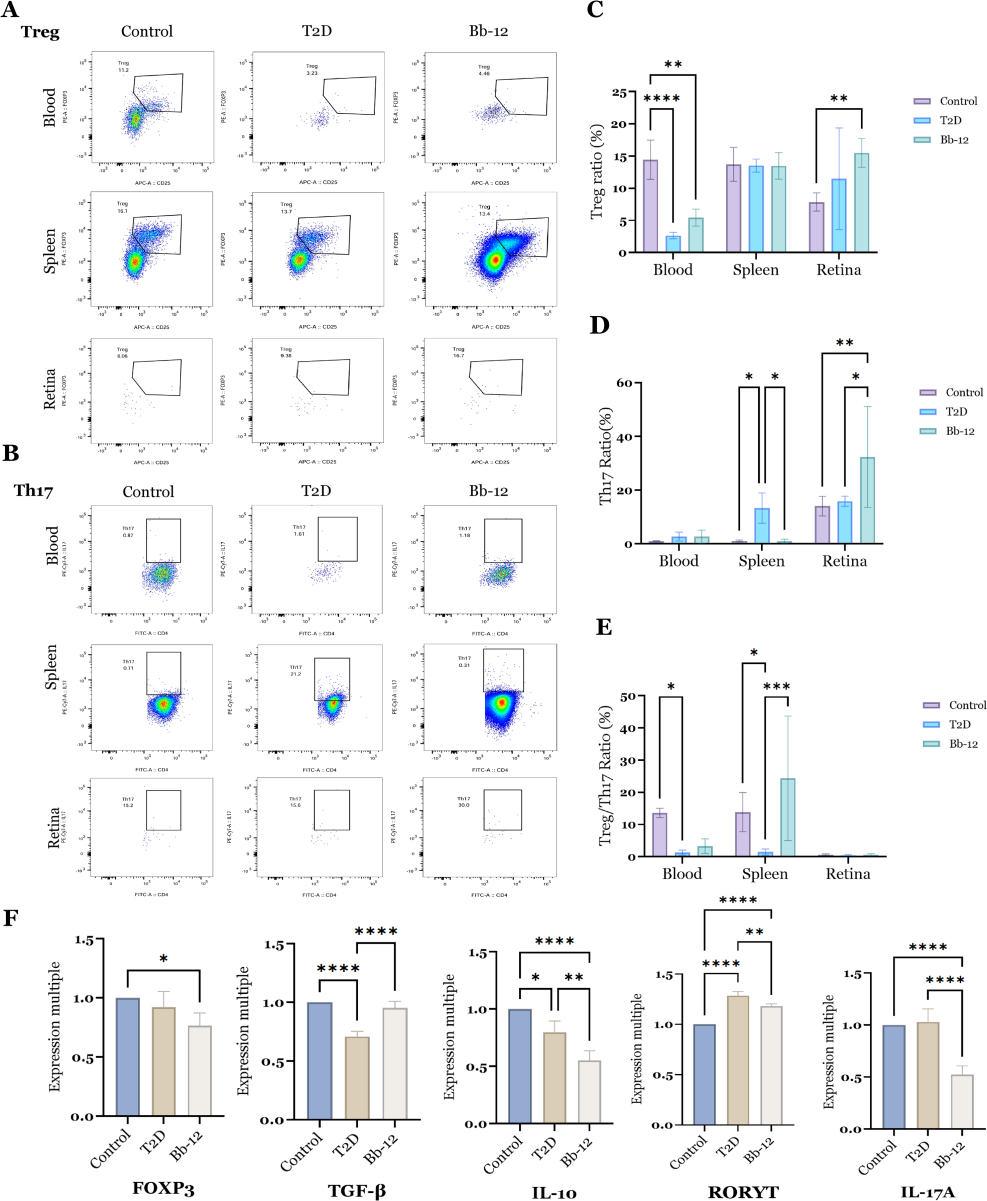
Flow cytometric analysis of Treg cells **(A)** and Th17 cells **(B)** in blood, spleen, and retina across the three groups, with statistical comparisons **(C–E)**; mRNA expression levels of Treg- and Th17-associated markers **(F)** (n=5). *P < 0.05, **P < 0.01, ***P < 0.001, ****P < 0.0001.

As shown in **Figures 4A–E**, the proportion of Tregs in peripheral blood was significantly reduced in diabetic mice compared to controls (P < 0.01). Bb-12 treatment partially restored Treg levels, although this change was not statistically significant. In splenic tissue, the T2D group exhibited significantly elevated Th17 cell percentages relative to control and Bb-12 groups (P < 0.05). In retinal tissue, the Bb-12 group showed increased proportions of both Treg and Th17 cells compared to controls (P < 0.01). However, due to the low number of T lymphocytes in retinal samples, flow cytometry data were prone to variability and thus interpreted cautiously.

To further characterize the dynamics of retinal Treg and Th17 cells, the mRNA expression of key markers was evaluated. *Foxp3*, the master regulator of Tregs, was downregulated in diabetic mice, while Bb-12 supplementation partially restored *TGF-β* expression. Interestingly, *IL-10* expression was reduced in the Bb-12 group relative to T2D. In contrast, the proinflammatory Th17-associated genes *RORγt* and *IL-17A* were significantly downregulated in the Bb-12 group.

In summary, *Bifidobacterium animalis subsp. lactis Bb-12* increased circulating Treg levels and modulated the splenic Treg/Th17 balance in diabetic mice. Although the low retinal cell yield limited the use of flow cytometry, the mRNA data indicate that Bb-12 suppresses retinal Th17-associated inflammation. (**Figure 4** about here)

### 3.7 Effects of bifidobacterium animalis subsp. lactis Bb-12 on gut microbiota in diabetic mice

**Figure 5:**
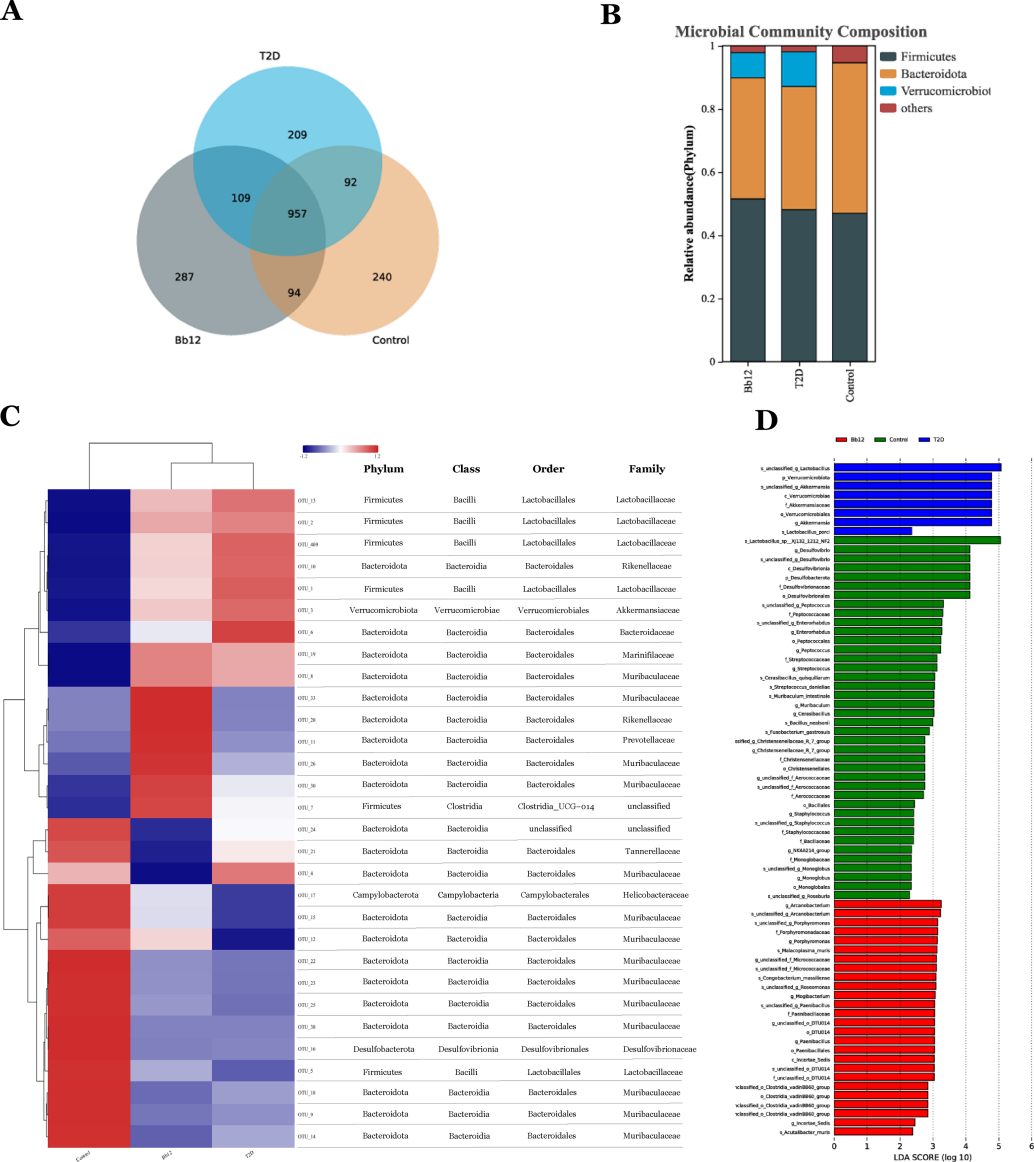
Venn diagram of species composition among the three groups **(A)** bar chart of microbial community composition **(B)** species abundance heatmap with hierarchical clustering **(C)** Linear discriminant analysis effect size (LEfSe) analysis **(D)** (n=8).

To assess microbial composition and diversity, sequencing reads were merged, quality-filtered, and clustered into OTUs after chimera removal. Representative OTU sequences were taxonomically annotated by comparison with reference databases. Subsequent analyses of microbial diversity and community structure were based on OTU units. The Venn diagram (**Figure 5A**) illustrates differences in species composition among groups: the control group harbored 240 unique OTUs, the T2D group 209, and the Bb-12 group 287. Shared OTUs numbered 92 between control and T2D, 94 between control and Bb-12, and 109 between Bb-12 and T2D groups. At the phylum and genus levels, bar plots (**Figure 5B**) revealed Firmicutes and Bacteroidota as dominant in controls. Diabetic mice showed reduced Bacteroidota and elevated Verrucomicrobiota, which Bb-12 partially reversed. The species abundance heatmap with hierarchical clustering (**Figure 5C**) revealed distinct microbial profiles between diabetic and control mice. The control group was characterized by 14 enriched OTUs (12 belonging to Bacteroidota), the Bb-12 group by 6 OTUs (5 belonging to Bacteroidota), and the T2D group by 7 OTUs (4xbelonging to Firmicutes). At the phylum, class, and order levels, the control and Bb-12 groups showed greater similarity. Linear discriminant analysis effect size (LEfSe) analysis (**Figure 5D**) identified taxa with significant differential abundance among groups; OTUs with LDA >3.0 were considered biomarkers. Six OTUs in controls and seven in T2D had LDA >4.0 (high-confidence), while Bb-12 had 20 OTUs with LDA >3.0 but none >4.0. (**Figure 5** about here)

**Figure 6:**
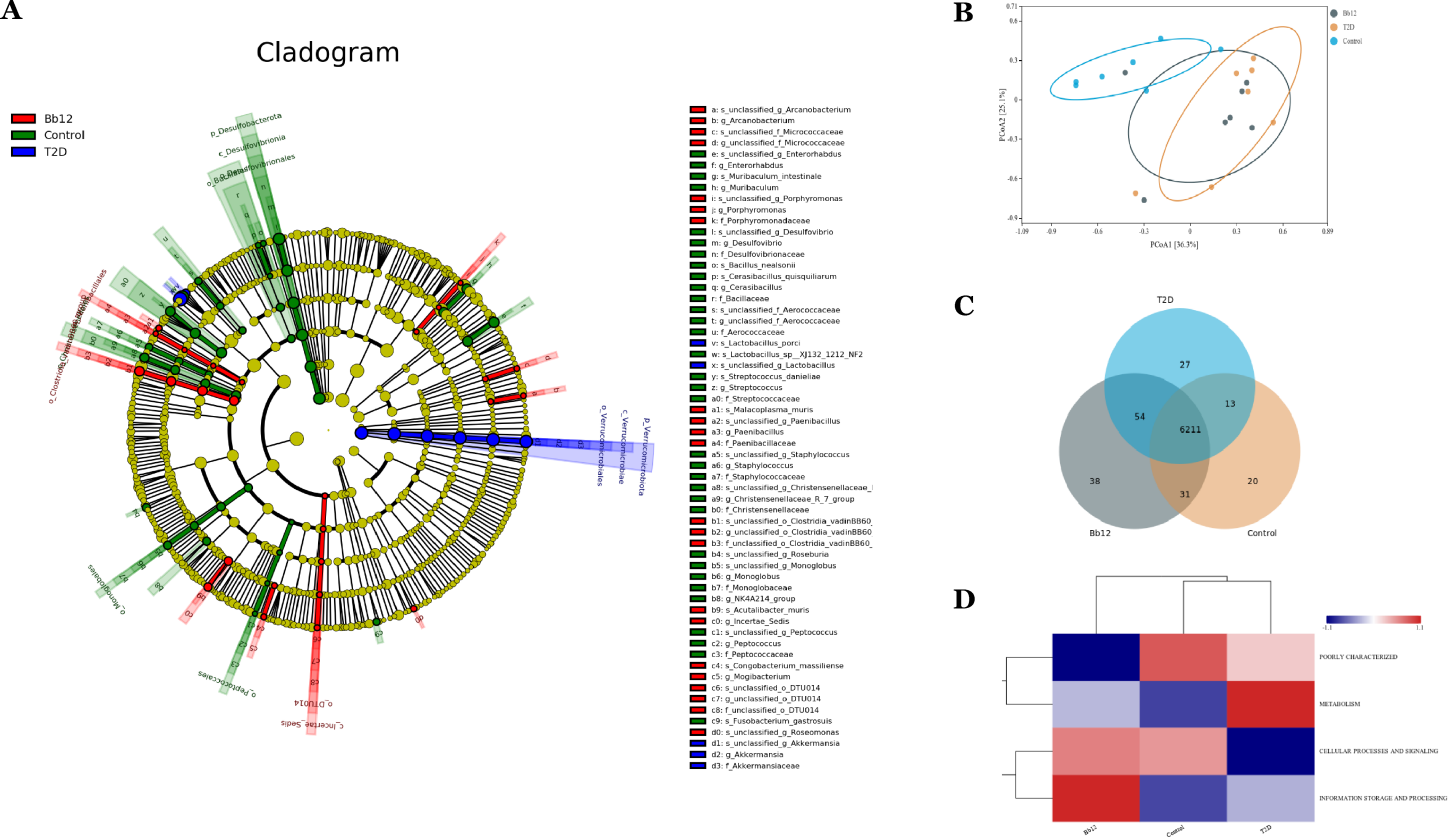
The cladogram generated from LEfSe analysis **(A)** principal coordinates analysis (PCoA) **(B)** functional pathway analysis based on the KEGG database **(C)** pathway clustering analysis **(D)** (n=8).

The cladogram (**Figure 6A**) illustrates phylogenetic relationships, showing closer taxonomic relatedness between the control and Bb-12 groups. Principal coordinates analysis (PCoA) based on OTU composition (**Figure 6B**) revealed greater similarity between the control and Bb-12 groups than between the control and T2D groups. Functional pathway analysis based on the KEGG database (**Figure 6C**) revealed 31 shared pathways between the control and Bb-12 groups, compared to 13 shared pathways between the control and T2D groups, and 54 shared pathways between the T2D and Bb-12 groups. Cluster analysis of functional categories from the COG database (**Figure 6D**) indicated that the Bb-12 group exhibited a relative enrichment in pathways related to information storage and processing compared to the other groups. (**Figure 6** about here)

## 4 Discussion

This study investigated whether *Bifidobacterium animalis subsp. lactis Bb-12* supplementation ameliorates diabetic phenotypes and retinal pathology, supporting the gut-retina axis and offering therapeutic insights. Our results show that Bb-12 mitigates obesity, hepatic steatosis, retinal vascular damage, and improved retinal function and gut microbiota composition.

As diabetes progresses, retinal microvascular damage—such as pericyte loss, disrupted cell junctions, and low-grade inflammation—becomes evident (Maurissen et al., 2024). This is closely linked to elevated intraocular VEGF levels, which drive neovascularization and form the basis for anti-VEGF therapies (Crabtree and Chang, 2021). Other emerging targets include IFN-γ-mediated PDGFRβ suppression (Dharmarajan et al., 2023) and the STING pathway (Liu et al., 2023) activation. The retina, although immune-privileged, becomes vulnerable to immune cell infiltration as DR advances (Zhao et al., 2024). The Treg/Th17 axis plays a central role in inflammation and immune imbalance (Zhang et al., 2021), with a known shift toward Th17 dominance in diabetes (Chen et al., 2016; Li L. et al., 2024). Th17 cells may penetrate the compromised blood-retinal barrier and promote DR via IL-17 (Mickael et al., 2025). Modulating this axis locally could slow DR progression, but current methods are often invasive (Wu et al., 2016). Our findings suggest the potential of non-invasive microbiota-based interventions to regulate intraocular inflammation, supporting future therapeutic strategies.

The human gut microbiota plays a crucial role in maintaining immune homeostasis and regulating the intestinal T lymphocyte population (Cristofori et al., 2021). For example, the symbiosis factor PSA produced by Bacteroides fragilis can directly activate TLR2 on Foxp3+ regulatory T cells, thereby promoting mucosal tolerance (Round et al., 2011). Combined administration of arginine and *Bifidobacterium animalis* subsp. *lactis* LKM512 elevates circulating and colonic polyamine levels, which is associated with reduced colonic TNF and IL-6 expression, thereby enhancing resistance to oxidative stress and extending lifespan (Rooks and Garrett, 2016). In a dextran sulfate sodium (DSS)-induced chronic colitis model, supplementation with Bifidobacterium adolescentis increased colonic lamina propria Treg and Th2 cells, suppressing excessive intestinal inflammation (Fan et al., 2021). Beyond local immune regulation, the gut microbiota also influences distant organs. For instance, the increased abundance of Akkermansia and Lactobacillus may alleviate anterior cruciate ligament transection-induced osteoarthritis via the “microbiota–gut–joint” axis and mitigate excessive bone loss (Deng et al., 2024). Intestinal alterations, such as mucosal injury and barrier disruption associated with nutrient malabsorption, have been linked to inflammatory responses following traumatic brain injury (TBI), with gut-targeted interventions showing potential to reduce neuroinflammation (El Baassiri et al., 2024).

Regarding the gut–retina axis, gut microbiota-derived metabolites and short-chain fatty acids (SCFAs) may help slow the progression of DR (Huang et al., 2023). SCFAs, as primary microbial metabolites, also act as ligands for G protein-coupled receptors; thus, dysbiosis can disrupt cellular homeostasis through altered signaling pathways (Ikeda et al., 2022). Jason Y. Zhang et al. further demonstrated that metformin modulates the gut microbiome to increase Bifidobacterium and Akkermansia abundance, downregulate proangiogenic genes (Tie1, Pgf, and Gata2), and attenuate the development of age-related macular degeneration (AMD) (Zhang JY et al., 2023). These findings underscore the therapeutic potential of systemic anti-inflammatory approaches and modulation of the gut microbiota in managing neovascular and chronic inflammatory ocular diseases (Baldi et al., 2024).

Clinical studies have reported a reduced abundance of Bacteroides fragilis in the gut microbiota of patients with diabetes (Sun et al., 2018; Letchumanan et al., 2022). Bacteroidetes-derived sphingolipids, including inositol phosphorylceramide and deoxysphingolipids, are negatively correlated with inflammation and inflammatory bowel disease (IBD) in humans (Brown et al., 2019). Ahmed et al. demonstrated that the Firmicutes-to-Bacteroidetes (F/B) ratio is positively associated with body mass index (BMI) (Ahmed et al., 2024), while Yang et al. found a similar positive correlation between the F/B ratio and hypertension (Yang et al., 2015). Dysregulation of this ratio has been closely linked to inflammatory diseases (Tsai et al., 2025). In our study, Bb-12 supplementation improved the F/B ratio in diabetic mice, potentially influencing fecal metabolite profiles and associated metabolic pathways, including pyrimidine, β-alanine, and purine metabolism (Li X et al., 2024), although the precise mechanisms remain unclear. This study further supports the existence and therapeutic relevance of the gut–retina axis, laying a foundation for future investigations and clinical trials. However, this study has limitations, our study lacks an investigation of the temporal relationship between gut microbiota alterations and immune modulation, as well as an exploration of microbial metabolites in relation to diabetic retinopathy DR and incomplete elucidation of the underlying pathways. Future research will aim to clarify these mechanisms in greater depth. We will further investigate the unique systemic regulatory mechanisms underlying the alleviation of DR, perform time-course analyses to closely monitor immune and microbiota dynamics, and conduct antibiotic depletion experiments to verify the essential role of gut microbiota in this regulatory process. Current evidence linking diabetic retinopathy to gut microbiota is largely derived from non-interventional cross-sectional studies. Given that various antidiabetic medications can differentially modulate gut microbial composition, our forthcoming clinical investigation will adopt more stringent inclusion criteria.

## 5 Conclusion

*Bifidobacterium animalis subsp. lactis Bb-12* ameliorated obesity-related phenotypes, delayed retinal vascular pathology, and improved visual function in diabetic mice. These effects may be associated with modulation of the Treg/Th17 axis and the F/B ratio. Collectively, these findings further substantiate the clinical relevance of the gut–retina axis and offer new perspectives for disease management.

## Data Availability

The original contributions presented in the study are included in the article/**Supplementary Material**. Further inquiries can be directed to the corresponding authors.

## References

[B1] Ahmed K. Choi H. N. Cho S. R. Yim J. E. (2024). Association of Firmicutes/Bacteroidetes ratio with body mass index in Korean type 2 diabetes mellitus patients. Metabolites. 14, 518. doi: 10.3390/metabo14100518, PMID: 39452900 PMC11509432

[B2] American Diabetes Association (2013). Diagnosis and classification of diabetes mellitus. Diabetes Care 36, S67–S74. doi: 10.2337/dc13-S067, PMID: 23264425 PMC3537273

[B3] Azushima K. Gurley S. Coffman T. (2018). Modelling diabetic nephropathy in mice. Nat Rev Nephrol. 14, 48–56. doi: 10.1038/nrneph.2017.142, PMID: 29062142

[B4] Bai J. Wan Z. Zhang Y. Wang T. Xue Y. Peng Q. (2022). Composition and diversity of gut microbiota in diabetic retinopathy. Front. Microbiol. 13. doi: 10.3389/fmicb.2022.926926, PMID: 36081798 PMC9445585

[B5] Baldi S. Pagliai G. Di Gloria L. Pallecchi M. Barca F. Pieri B. . (2024). Beneficial effects of micronutrient supplementation in restoring the altered microbiota and gut-retina axis in patients with neovascular age-related macular degeneration-a randomized clinical trial. Nutrients. 16, 3971. doi: 10.3390/nu16223971, PMID: 39599758 PMC11597754

[B6] Barrio C. Arias-Sánchez S. Martín-Monzón I. (2022). The gut microbiota-brain axis, psychobiotics and its influence on brain and behavior: a systematic review. Psychoneuroendocrinology. 137, 105640. doi: 10.1016/j.psyneuen.2021.105640, PMID: 34942539

[B7] Brown D. M. Wykoff C. C. Boyer D. Heier J. S. Clark W. L. Emanuelli A. . (2021). Evaluation of intravitreal aflibercept for the treatment of severe nonproliferative diabetic retinopathy: results from the PANORAMA randomized clinical trial. JAMA Ophthalmol. 139, 946–955. doi: 10.1001/jamaophthalmol.2021.2809, PMID: 34351414 PMC8343518

[B8] Brown E. M. Ke X. Hitchcock D. Jeanfavre S. Avila-Pacheco J. Nakata T. . (2019). Bacteroides-derived sphingolipids are critical for maintaining intestinal homeostasis and symbiosis. Cell Host Microbe 25, 668–680.e7. doi: 10.1016/j.chom.2019.04.002, PMID: 31071294 PMC6544385

[B9] Chan K. Hoon M. Pattnaik B. R. Ver Hoeve J. N. Wahlgren B. Gloe S. . (2020). Vigabatrin-induced retinal functional alterations and second-order neuron plasticity in C57BL/6J mice. Invest. Ophthalmol. Vis. Sci. 61, 17. doi: 10.1167/iovs.61.2.17, PMID: 32053727 PMC7326505

[B10] Chen H. Ren X. Liao N. Wen F. (2016). Th17 cell frequency and IL-17A concentrations in peripheral blood mononuclear cells and vitreous fluid from patients with diabetic retinopathy. J. Int. Med. Res. 44, 1403–1413. doi: 10.1177/0300060516672369, PMID: 27885039 PMC5536736

[B11] Crabtree G. S. Chang J. S. (2021). Management of complications and vision loss from proliferative diabetic retinopathy. Curr. Diab. Rep. 21, 33. doi: 10.1007/s11892-021-01396-2, PMID: 34477996

[B12] Cristofori F. Dargenio V. N. Dargenio C. Miniello V. L. Barone M. Francavilla R. (2021). Anti-inflammatory and immunomodulatory effects of probiotics in gut inflammation: a door to the body. Front. Immunol. 12. doi: 10.3389/fimmu.2021.578386, PMID: 33717063 PMC7953067

[B13] Deng Z. Yang C. Xiang T. Dou C. Sun D. Dai Q. . (2024). Gold nanoparticles exhibit anti-osteoarthritic effects via modulating interaction of the “microbiota-gut-joint” axis. J. Nanobiotechnology. 22, 157. doi: 10.1186/s12951-024-02447-y, PMID: 38589904 PMC11000357

[B14] Dharmarajan S. Carrillo C. Qi Z. Wilson J. M. Baucum A. J. Sorenson C. M. . (2023). Retinal inflammation in murine models of type 1 and type 2 diabetes with diabetic retinopathy. Diabetologia. 66, 2170–2185. doi: 10.1007/s00125-023-05995-4, PMID: 37670018 PMC10541343

[B15] El Baassiri M. G. Raouf Z. Badin S. Escobosa A. Sodhi C. P. Nasr I. W. (2024). Dysregulated brain-gut axis in the setting of traumatic brain injury: review of mechanisms and anti-inflammatory pharmacotherapies. J. Neuroinflammation. 21, 124. doi: 10.1186/s12974-024-03118-3, PMID: 38730498 PMC11083845

[B16] Erttmann S. F. Swacha P. Aung K. M. Brindefalk B. Jiang H. Härtlova A. . (2022). The gut microbiota prime systemic antiviral immunity via the cGAS-STING-IFN-I axis. Immunity 55, 847–861.e10. doi: 10.1016/j.immuni.2022.04.006, PMID: 35545033

[B17] Fan L. Qi Y. Qu S. Chen X. Li A. Hendi M. . (2021). Adolescentis ameliorates chronic colitis by regulating Treg/Th2 response and gut microbiota remodeling. Gut Microbes 13, 1–17. doi: 10.1080/19490976.2020.1826746, PMID: 33557671 PMC7889144

[B18] Hashida N. Nishida K. (2023). Recent advances and future prospects: current status and challenges of the intraocular injection of drugs for vitreoretinal diseases. Adv. Drug Delivery Rev. 198, 114870. doi: 10.1016/j.addr.2023.114870, PMID: 37172783

[B19] Huang Y. Wang Z. Ma H. Ji S. Chen Z. Cui Z. . (2021). Dysbiosis and implication of the gut microbiota in diabetic retinopathy. Front. Cell. Infect. Microbiol. 11. doi: 10.3389/fcimb.2021.646348, PMID: 33816351 PMC8017229

[B20] Huang Y. Wang Z. Ye B. Ma J. H. Ji S. Sheng W. . (2023). Sodium butyrate ameliorates diabetic retinopathy in mice via the regulation of gut microbiota and related short-chain fatty acids. J. Transl. Med. 21, 451. doi: 10.1186/s12967-023-04259-4, PMID: 37420234 PMC10329333

[B21] Ikeda T. Nishida A. Yamano M. Kimura I. (2022). Short-chain fatty acid receptors and gut microbiota as therapeutic targets in metabolic, immune, and neurological diseases. Pharmacol. Ther. 239, 108273. doi: 10.1016/j.pharmthera.2022.108273, PMID: 36057320

[B22] Jiao Y. Wu L. Huntington N. D. Zhang X. (2020). Crosstalk between gut microbiota and innate immunity and its implication in autoimmune diseases. Front. Immunol. 11. doi: 10.3389/fimmu.2020.00282, PMID: 32153586 PMC7047319

[B23] Kammoun S. Rekik M. Dlensi A. Aloulou S. Smaoui W. Sellami S. . (2024). The gut-eye axis: the retinal/ocular degenerative diseases and the emergent therapeutic strategies. Front. Cell. Neurosci. 18. doi: 10.3389/fncel.2024.1468187, PMID: 39391760 PMC11464360

[B24] Letchumanan G. Abdullah N. Marlini M. Baharom N. Lawley B. Omar M. R. . (2022). Gut microbiota composition in prediabetes and newly diagnosed type 2 diabetes: a systematic review of observational studies. Front. Cell. Infect. Microbiol. 12. doi: 10.3389/fcimb.2022.943427, PMID: 36046745 PMC9422273

[B25] Li X. Chen Y. Peng X. Zhu Y. Duan W. Ji R. . (2024). Anti-inflammation mechanisms of a homogeneous polysaccharide from Phyllanthus emblica L. @ on DSS induced colitis mice via the gut microbiota and metabolites alteration. Food Chem. 459, 140346. doi: 10.1016/j.foodchem.2024.140346, PMID: 38981378

[B26] Li L. Chen J. Wang Z. Xu Y. Yao H. Lei W. . (2024). NECA alleviates inflammatory responses in diabetic retinopathy through dendritic cell toll-like receptor signaling pathway. Front. Immunol. 15. doi: 10.3389/fimmu.2024.1415004, PMID: 38895119 PMC11182989

[B27] Liu H. Ghosh S. Vaidya T. Bammidi S. Huang C. Shang P. . (2023). Activated cGAS/STING signaling elicits endothelial cell senescence in early diabetic retinopathy. JCI Insight 8, e168945. doi: 10.1172/jci.insight.168945, PMID: 37345657 PMC10371250

[B28] Maurissen T. L. Spielmann A. J. Schellenberg G. Bickle M. Vieira J. R. Lai S. Y. . (2024). Modeling early pathophysiological phenotypes of diabetic retinopathy in a human inner blood-retinal barrier-on-a-chip. Nat. Commun. 15, 1372. doi: 10.1038/s41467-024-45456-z, PMID: 38355716 PMC10866954

[B29] McAnany J. J. Persidina O. S. Park J. C. (2022). Clinical electroretinography in diabetic retinopathy: a review. Surv. Ophthalmol. 67, 712–722. doi: 10.1016/j.survophthal.2021.08.011, PMID: 34487740 PMC9158180

[B30] Merenstein D. J. Tan T. P. Molokin A. Smith K. H. Roberts R. F. Shara N. M. . (2015). Safety of Bifidobacterium animalis subsp. lactis (B. lactis) strain BB-12-supplemented yogurt in healthy adults on antibiotics: a phase I safety study. Gut Microbes 6, 66–77. doi: 10.1080/19490976.2015.1005484, PMID: 25569274 PMC4615198

[B31] Mickael M. E. Kubick N. Miftari K. Horbańczuk J. O. Atanasov A. G. Binçe K. . (2025). The role of Th17/Treg axis in retinal pathology associated with diabetes and treatment options. Biology. 14, 275. doi: 10.3390/biology14030275, PMID: 40136531 PMC11940215

[B32] Min B. H. Devi S. Kwon G. H. Gupta H. Jeong J. J. Sharma S. P. . (2024). Gut microbiota-derived indole compounds attenuate metabolic dysfunction-associated steatotic liver disease by improving fat metabolism and inflammation. Gut Microbes 16, 2307568. doi: 10.1080/19490976.2024.2307568, PMID: 38299316 PMC10841017

[B33] Nocerino R. De Filippis F. Cecere G. Marino A. Micillo M. Di Scala C. . (2020). The therapeutic efficacy of Bifidobacterium animalis subsp. lactis BB-12^®^ in infant colic: a randomized, double blind, placebo-controlled trial. Aliment. Pharmacol. Ther. 51, 110–120. doi: 10.1111/apt.15561, PMID: 31797399 PMC6973258

[B34] Parker A. Romano S. Ansorge R. Aboelnour A. Le Gall G. Savva G. M. . (2022). Fecal microbiota transfer between young and aged mice reverses hallmarks of the aging gut, eye, and brain. Microbiome. 10, 68. doi: 10.1186/s40168-022-01243-w, PMID: 35501923 PMC9063061

[B35] Qin X. Shi H. Li H. Chu B. Zhang J. Wen Z. . (2025). Wearable electrodriven switch actively delivers macromolecular drugs to fundus in non-invasive and controllable manners. Nat. Commun. 16, 33. doi: 10.1038/s41467-024-55336-1, PMID: 39747871 PMC11695998

[B36] Rooks M. G. Garrett W. S. (2016). Gut microbiota, metabolites and host immunity. Nat. Rev. Immunol. 16, 341–352. doi: 10.1038/nri.2016.42, PMID: 27231050 PMC5541232

[B37] Round J. L. Lee S. M. Li J. Tran G. Jabri B. Chatila T. A. . (2011). The toll-like receptor 2 pathway establishes colonization by a commensal of the human microbiota. Science. 332, 974–977. doi: 10.1126/science.1206095, PMID: 21512004 PMC3164325

[B38] Rowan S. Taylor A. (2018). Gut microbiota modify risk for dietary glycemia-induced age-related macular degeneration. Gut Microbes. 9, 452–457. doi: 10.1080/19490976.2018.1435247, PMID: 29431583 PMC6219648

[B39] Semeraro F. Morescalchi F. Cancarini A. Russo A. Rezzola S. Costagliola C. (2019). Diabetic retinopathy, a vascular and inflammatory disease: therapeutic implications. Diabetes Metab. 45, 517–527. doi: 10.1016/j.diabet.2019.04.002, PMID: 31005756

[B40] Sun L. Xie C. Wang G. Wu Y. Wu Q. Wang X. . (2018). Gut microbiota and intestinal FXR mediate the clinical benefits of metformin. Nat. Med. 24, 1919–1929. doi: 10.1038/s41591-018-0222-4, PMID: 30397356 PMC6479226

[B41] Tonucci L. B. Olbrich Dos Santos K. M. Licursi de Oliveira L. Rocha Ribeiro S. M. Duarte Martino H. S. (2017). Clinical application of probiotics in type 2 diabetes mellitus: a randomized, double-blind, placebo-controlled study. Clin. Nutr. (edinb Scotl). 36, 85–92. doi: 10.1016/j.clnu.2015.11.011, PMID: 26732026

[B42] Tsai Y. C. Tai W. C. Liang C. M. Wu C. K. Tsai M. C. Hu W. H. . (2025). Alternations of the gut microbiota and the Firmicutes/Bacteroidetes ratio after biologic treatment in inflammatory bowel disease. J. Microbiol. Immunol. Infect. 58, 62–69. doi: 10.1016/j.jmii.2024.09.006, PMID: 39393964

[B43] Wu W. He Z. Zhang Z. Yu X. Song Z. Li X. (2016). Intravitreal injection of rapamycin-loaded polymeric micelles for inhibition of ocular inflammation in rat model. Int. J. Pharm. 513, 238–246. doi: 10.1016/j.ijpharm.2016.09.013, PMID: 27609662

[B44] Yang T. Santisteban M. M. Rodriguez V. Li E. Ahmari N. Carvajal J. M. . (2015). Gut dysbiosis is linked to hypertension. Hypertension. 65, 1331–1340. doi: 10.1161/HYPERTENSIONAHA.115.05315, PMID: 25870193 PMC4433416

[B45] Yourick M. R. Sandkam B. A. Gammerdinger W. J. Escobar-Camacho D. Nandamuri S. P. Clark F. E. . (2019). Diurnal variation in opsin expression and common housekeeping genes necessitates comprehensive normalization methods for quantitative real-time PCR analyses. Mol. Ecol. Resour. 19, 1447–1460. doi: 10.1111/1755-0998.13062, PMID: 31325910 PMC6995727

[B46] Zhang S. Gang X. Yang S. Cui M. Sun L. Li Z. . (2021). The alterations in and the role of the Th17/Treg balance in metabolic diseases. Front. Immunol. 12. doi: 10.3389/fimmu.2021.678355, PMID: 34322117 PMC8311559

[B47] Zhang Y. Wang T. Wan Z. Bai J. Xue Y. Dai R. . (2023). Alterations of the intestinalmicrobiota in age-related macular degeneration. Front. Microbiol. 14. doi: 10.3389/fmicb.2023.1069325, PMID: 37089564 PMC10113553

[B48] Zhang J. Y. Xiao J. Xie B. Barba H. Boachie-Mensah M. Shah R. N. . (2023). Oral metformin inhibits choroidal neovascularization by modulating the gut-retina axis. Invest. Ophthalmol. Vis. Sci. 64, 21. doi: 10.1167/iovs.64.15.21, PMID: 38108689 PMC10732090

[B49] Zhao B. Zhao Y. Sun X. (2024). Mechanism and therapeutic targets of circulating immune cells in diabetic retinopathy. Pharmacol. Res. 210, 107505. doi: 10.1016/j.phrs.2024.107505, PMID: 39547465

