# Supplementary material for: Bifidobacterium animalis subsp. lactis BB-12 attenuates diabetic retinopathy through gut microbiota modulation: evidence for the gut–retinal axis
Source: Front Cell Infect Microbiol. 2025 Nov 6;15:1681943. doi: 10.3389/fcimb.2025.1681943 (PMC12631188; doi:10.3389/fcimb.2025.1681943)
Supplement: Supplementary file 1 [file Table1.docx]

Supplementary Files

Due to the word limit of the main text, the method part is expanded in detail in this section.

2.1 Animal experiment and study design

In this animal study, male C57BLKS/J db/db mice with spontaneous mutations in the leptin receptor and their littermate controls (SPF grade) were used (purchased from Jiangsu Jicui Laboratory Animal Co., Ltd.). Animals were randomly assigned to three groups (Control group, T2D group, and Bb-12 group), with nine mice per group. The Control group consisted of male C57BLKS/J db/m heterozygous littermates of db/db mice, serving as non-diabetic controls, as these mice do not exhibit type 2 diabetes–related phenotypes. The T2D group comprised male homozygous db/db mice, which develop spontaneous diabetes and morbid obesity due to leptin receptor gene deficiency. Compared to chemically induced diabetic models, this genetic model more closely resembles the chronic insulin metabolic dysregulation characteristic of human type 2 diabetes. The Bb-12 group served as the intervention cohort; these diabetic mice received a daily oral gavage of 0.1 mL of *Bifidobacterium animalis* subsp. *lactis* Bb-12 probiotic suspension (purchased from Shanghai Difule Biotechnology Co., Ltd.; 1.9 × 10¹⁰ CFU/mL).

All experimental procedures were conducted in strict accordance with the Guidelines for the Use of Laboratory Animals issued by the Chinese Association for Laboratory Animal Science and were approved by the Institutional Animal Care and Use Committee of the Third Affiliated Hospital of Sun Yat-sen University. All mice were housed in individually ventilated cages under controlled conditions (temperature 22 ± 1 °C, humidity 50 ± 5%) with ad libitum access to food and water. Bedding was changed daily in the T2D and Bb-12 groups. The Control group received no specific treatment. The T2D group was administered a daily oral gavage of 0.1 mL sterile saline, while the Bb-12 group received 0.1 mL *Bifidobacterium animalis* subsp. *lactis* Bb-12 suspension. The experimental period lasted 12 weeks. Throughout the study, fasting body weight and fasting blood glucose were recorded biweekly, and water intake and general activity were closely monitored.

2.2 Retina optic coherence tomography (OCT)

At week 12 of the experiment, the mice were anesthetized with an intraperitoneal injection of sodium pentobarbital (40 mg/kg). Pupillary dilation was induced using compound tropicamide eye drops. Once adequate mydriasis was confirmed, retinal optical coherence tomography (OCT) imaging was performed with equipment from Heidelberg Engineering (Germany). Fundus photographs and OCT images were recorded, and retinal layer analysis was later conducted using ImageJ software.

2.3 Electroretinography (ERG)

At week 12, retinal ERG was performed to evaluate retinal electrophysiological function. Mice were dark-adapted for 12 hours before the procedure. Anesthesia and pupillary dilation were conducted as described above. Following dilation, sodium carboxymethylcellulose eye drops were applied to the cornea as a coupling agent and to protect the ocular surface. Ring-shaped corneal electrodes were gently placed in contact with the corneal surfaces bilaterally. Reference electrodes were inserted subcutaneously into the cheeks, and a ground electrode was placed subcutaneously in the tail^10^. Under scotopic conditions, stimuli of 0.0003 cd·s/m², 0.01 cd·s/m², and 3.0 cd·s/m² were sequentially applied. After 5 minutes of light adaptation, a photopic stimulus of 3.0 cd·s/m² was delivered. ERG waveforms were recorded and subsequently analyzed using Spyder (Python 3.12).

2.4 Retinal Whole Mounts

An appropriate amount of Evans Blue powder (Macklin Biochemical, Shanghai) was thoroughly dissolved in sterile saline and then filtered through a 70 µm cell strainer. After restraining the mice, the tail veins were dilated with ethanol, and the Evans Blue solution was administered via tail vein injection. Changes in coloration of the limbs and head were monitored following injection. After allowing the dye to circulate for 1 hour, mice were euthanized by overdose of sodium pentobarbital. The eyeballs were enucleated and fixed in 4% paraformaldehyde at 4 °C for 12 hours. Fixed eyes were rinsed with PBS, and retinas were carefully dissected under a stereomicroscope. Retinas were flat-mounted onto glass slides and coverslipped. Images were acquired using a laser scanning confocal microscope (ZEISS LSM 880). Retinal vascular density was subsequently analyzed using Spyder (Python 3.12).

2.5 Hematoxylin–eosin (HE) and Periodic acid–Schiff (PAS) staining

At week 12, mice were euthanized, and the eyeballs, liver, and small intestine were collected and fixed in 4% paraformaldehyde. Tissues were dehydrated through a graded ethanol series, embedded in paraffin, and sectioned. HE staining was performed using standard protocols. Morphological evaluation was conducted with CaseViewer software. For retinal vascular analysis, periodic acid–Schiff (PAS) staining was performed. Anesthesia, tissue collection, and embedding procedures were the same as described above. Retinal sections were incubated in periodic acid solution at room temperature, rinsed with distilled water to remove residual reagent, and then immersed in Schiff reagent protected from light. After staining, sections were washed under running water and counterstained with hematoxylin. Following mounting, retinal vasculature was examined using CaseViewer software.

2.6 Flow cytometry of blood, spleen, and retina

Mice were euthanized via overdose anesthesia, followed by cardiac puncture to collect blood samples. Whole blood was collected into EDTA tubes, thoroughly mixed, and incubated in culture tubes before the addition of red blood cell lysis buffer to obtain a single-cell suspension. Spleen and retinal tissues were mechanically dissociated and filtered to prepare single-cell suspensions. Cells were stained for surface markers using APC/Cyanine7 CD45 Antibody, PerCP/Cyanine5.5 CD3 Antibody, APC CD25 Antibody, and FITC CD4 Antibody. Following fixation and permeabilization according to the manufacturer's protocols, intracellular staining was performed using PE FOXP3 Antibody and PE/Cyanine7 IL-17A Antibody (both from BioLegend, USA). Cell analysis was conducted using a Beckman Coulter CytoFLEX flow cytometer, and data were analyzed with FlowJo v11 software.

2.7 Retinal Quantitative Real-time PCR (qRCR)

Mice were euthanized by overdose anesthesia, and eyeballs were enucleated to isolate retinas. Total RNA was extracted following the manufacturer’s protocol using RNA extraction reagent, chloroform substitute, isopropanol, and RNA dissolution buffer. Reverse transcription was performed using a commercial cDNA synthesis kit on a PCR instrument. qPCR was conducted with Universal Blue SYBR Green qPCR Master Mix and specific gene primers. The amplification protocol consisted of an initial denaturation step at 95°C for 30 seconds, followed by 40 cycles of denaturation at 95°C for 15 seconds and extension at 60°C for 30 seconds, concluding with a melt curve analysis. Relative gene expression was calculated using the ΔΔCT method^11^. All reagents were purchased from Wuhan Seville Biotechnology.

| Gene name | Forward primer | Reverse primer |
| --- | --- | --- |
| RORYT | GGATGAGATTGCCCTCTACACG | GCGGCTTGGACCACGATG |
| IL-17 | TCCACCGCAATGAAGACCCT | CATGTGGTGGTCCAGCTTTCC |
| Foxp3 | ATGAGAAAGGCAAGGCCCAGT | GTGGCTACGATGCAGCAAGA |
| TGF-beta | GCTGAACCAAGGAGACGGAATA | GGCTGATCCCGTTGATTTCC |
| VEGF | GAGCGTTCACTGTGAGCCTTGT | TTAACTCAAGCTGCCTCGCCT |
| IL-10 | AATAAGCTCCAAGACCAAGGTGT | CATCATGTATGCTTCTATGCAGTTG |

2.8 Determination of blood biochemical indicators

Mice were euthanized by overdose anesthesia, followed by cardiac puncture to collect blood samples. Blood was centrifuged at 3000 rpm for 15 minutes at 4°C, and the serum was collected. Serum levels of aminotransferase (AST), alanine aminotransferase (ALT), triglycerides (TG), total cholesterol (TC), low-density lipoprotein cholesterol (LDL-C), and high-density lipoprotein cholesterol (HDL-C) were measured using the CASTS-DL0086 fully automated biochemical analyzer.

2.9 Gut microbiome analysis

Microbial DNA from mouse fecal samples was extracted using the E.Z.N.A.® Gel Extraction Kit (Omega Bio-tek, Norcross, GA, USA) following the manufacturer’s protocol. Subsequent library preparation was performed according to the NEBNext® Ultra™ DNA Library Prep Kit for Illumina® standard protocol. The V3–V4 regions of the 16S rRNA gene were PCR-amplified using primers 338F (5′-ACTCCTACGGGAGGCAGCA-3′) and 806R (5′-GGACTACHVGGGTWTCTAAT-3′). PCR conditions included an initial denaturation at 98°C for 30 seconds, followed by 15 cycles of denaturation at 98°C for 10 seconds, annealing at 65°C for 75 seconds, and extension at 65°C for 5 minutes. Amplicons were purified using NEBNext Sample Purification Beads. Library sequencing was performed by Megagen Biotechnology Co., Ltd. (Shenzhen, China) using the Illumina NovaSeq PE250 platform.


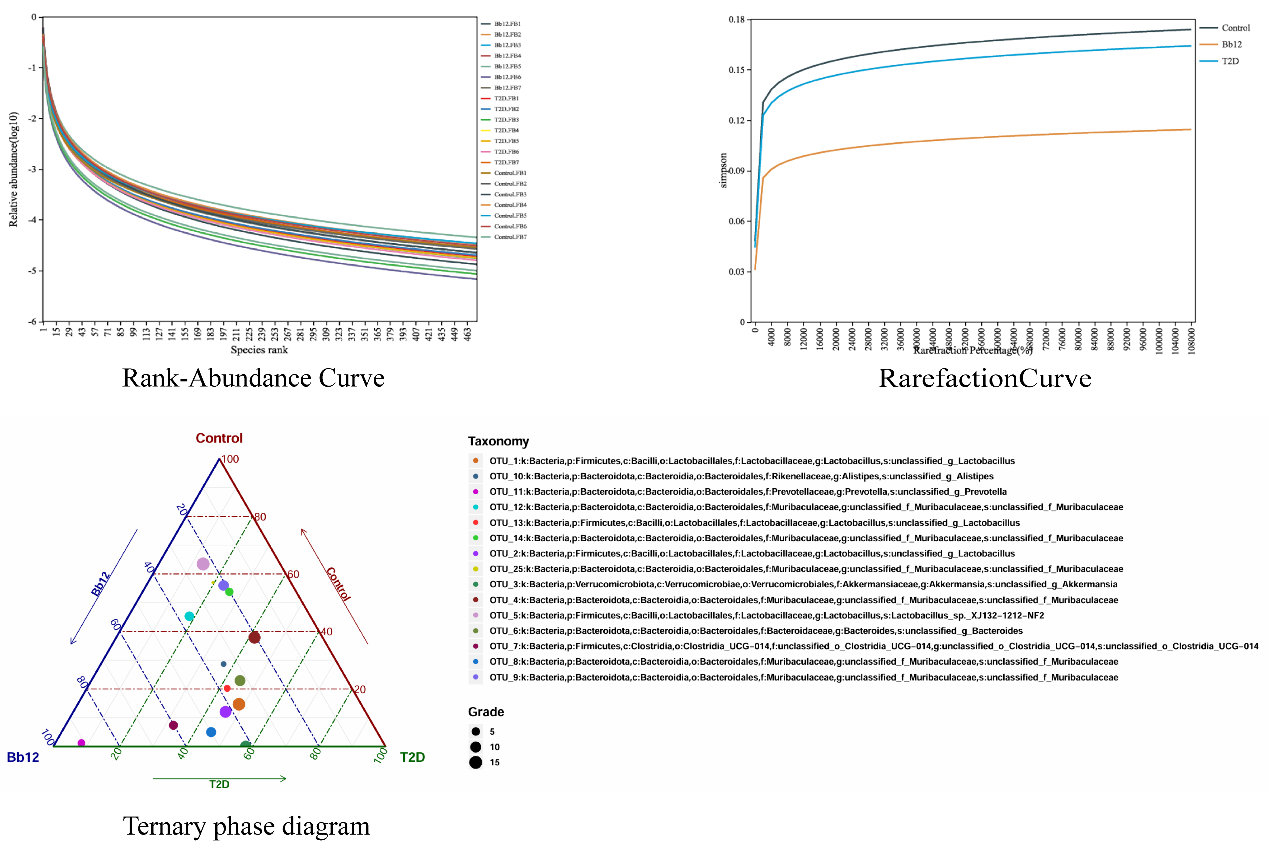


Figure S1: Rank-Abundance Curve, Rarefaction Curve and Ternary phase diagram of intestinal flora.
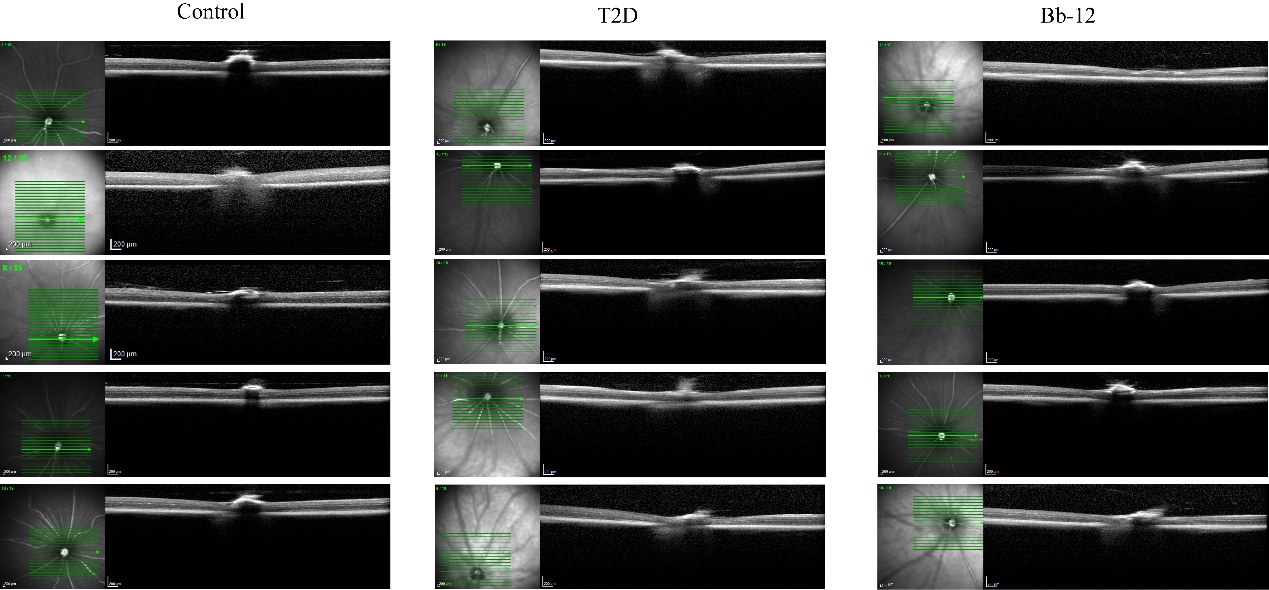


Figure S2: Typical OCT images.
